# ECOA-5. Integrative 3D spatial characterization of genomic and epigenomic intratumoral heterogeneity in glioblastoma

**DOI:** 10.1093/noajnl/vdab070.005

**Published:** 2021-07-05

**Authors:** Radhika Mathur, Qixuan Wang, Patrick Schupp, Stephanie Hilz, Chibo Hong, Ivan Smirnov, Marisa Lafontaine, Devika Nair, Sriranga Iyyanaki, Joanna Phillips, Susan Chang, Yan Li, Janine Lupo, Michael Oldham, Feng Yue, Joseph Costello

**Affiliations:** 1 University of California San Francisco, San Francisco, CA, USA; 2 Northwestern University, Evanston, IL, USA

## Abstract

Treatment failure in glioblastoma is often attributed to intratumoral heterogeneity (ITH), which fosters tumor evolution and selection of therapy-resistant clones. While genomic alterations are known contributors to ITH, emerging studies highlight functional roles for epigenomic ITH which integrates differentiation status, stochastic events, and microenvironmental inputs. Here, we have established a novel platform for integrative characterization of genomic and epigenomic ITH of glioblastoma in three-dimensional (3-D) space. In collaboration with neurosurgeons and biomedical imaging experts, we utilize 3-D surgical neuro-navigation to safely acquire ~10 tumor samples per patient representing maximal anatomical diversity. We conduct whole-exome sequencing, RNA sequencing, and assay for transposase-accessible chromatin using sequencing (ATAC-Seq) on each sample. The spatial location of each sample is mapped by its 3-D coordinates, allowing 360-degree visualization of genomic and epigenomic ITH for each patient. We demonstrate this approach on 8 patients with primary IDH-WT glioblastoma (83 spatially mapped samples), providing unprecedented insight into their spatial organization at the genomic and epigenomic levels. We link genetically defined tumor subclones to patterns of open chromatin and gene regulation, revealing underlying transcription factor binding at active promoters and enhancers. We also identify ITH in whole-genome doubling and focal oncogene amplification events in multiple patients, which we then link with epigenomic ITH. Further, to study microenvironmental inputs and their contribution to epigenomic ITH, we conduct deconvolution of RNA sequencing and ATAC-Seq data by analyzing feature co-variation. We resolve the 3-D spatial organization of immune, neural, and other nontumor cell types present in glioblastoma, characterizing their functional states and interactions with tumor cells. This work provides the most comprehensive spatial characterization of genomic and epigenomic ITH to date in glioblastoma. As a resource for further investigation, we have developed an interactive data sharing platform – The 3D Glioma Atlas – that enables 360-degree visualization of both genomic and epigenomic ITH.

